# Translational and oncologic significance of tertiary lymphoid structures in pancreatic adenocarcinoma

**DOI:** 10.3389/fimmu.2024.1324093

**Published:** 2024-02-01

**Authors:** Zachary Gao, Joseph Azar, Huili Zhu, Sophia Williams-Perez, Sung Wook Kang, Celia Marginean, Mark P. Rubinstein, Shalini Makawita, Hyun-Sung Lee, E. Ramsay Camp

**Affiliations:** ^1^ Michael E. DeBakey Department of Surgery, Baylor College of Medicine, Houston, TX, United States; ^2^ The Pelotonia Institute for Immuno-Oncology, Ohio State University Comprehensive Cancer Center, Columbus, OH, United States; ^3^ Dan L. Duncan Comprehensive Cancer Center, Baylor College of Medicine, Houston, TX, United States; ^4^ Systems Onco-Immunology Laboratory, David J. Sugarbaker Division of Thoracic Surgery, Michael E. DeBakey Department of Surgery, Baylor College of Medicine, Houston, TX, United States; ^5^ Baylor College of Medicine, Michael E. DeBakey VA Medical Center, Houston, TX, United States

**Keywords:** tertiary lymphoid structures, pancreatic adenocarcinoma, pancreatic cancer, translational ability, immune microenvironment

## Abstract

Pancreatic adenocarcinoma (PDAC) is an aggressive tumor with poor survival and limited treatment options. PDAC resistance to immunotherapeutic strategies is multifactorial, but partially owed to an immunosuppressive tumor immune microenvironment (TiME). However, the PDAC TiME is heterogeneous and harbors favorable tumor-infiltrating lymphocyte (TIL) populations. Tertiary lymphoid structures (TLS) are organized aggregates of immune cells that develop within non-lymphoid tissue under chronic inflammation in multiple contexts, including cancers. Our current understanding of their role within the PDAC TiME remains limited; TLS are complex structures with multiple anatomic features such as location, density, and maturity that may impact clinical outcomes such as survival and therapy response in PDAC. Similarly, our understanding of methods to manipulate TLS is an actively developing field of research. TLS may function as anti-tumoral immune niches that can be leveraged as a therapeutic strategy to potentiate both existing chemotherapeutic regimens and potentiate future immune-based therapeutic strategies to improve patient outcomes. This review seeks to cover anatomy, relevant features, immune effects, translational significance, and future directions of understanding TLS within the context of PDAC.

## 1 Introduction

Although pancreatic adenocarcinoma (PDAC) accounts for approximately 3% of all cancer in the US, it is projected to become the 2nd leading cause of cancer-related deaths in the US by 2030 (1). The prognosis for patients with PDAC remains poor with <10% five-year overall survival and modest survival benefit from standard treatment regimens (2). Immunotherapy has changed the paradigm in modern oncologic therapy for cancers such as melanoma and non-small cell lung cancer (3–6). Unfortunately, immune checkpoint inhibitor (ICI) therapy has proven ineffective for PDAC outside of the rare few with high microsatellite instability (7–12). Additionally, other strategies such as TCR therapy, CAR-T therapy, tumor-infiltrating lymphocyte (TIL) therapy, and mRNA vaccines are still in their nascency (13–15).

Immunotherapy failure has partially been attributed to the immunosuppressive tumor immune microenvironment (TiME) of PDAC (16). The PDAC TiME is complex and significant tumor immune heterogeneity exists both within the tumor, the peritumoral microenvironment, and across patients (17–20). The cellular constituents of the PDAC TiME consists of multiple immunosuppressive cell lines, including regulatory T cells (Tregs), myeloid-derived suppressor cells (MDSCs), and tumor-associated macrophages (TAMs) (16, 21–30). These features of the TiME contribute to PDAC tumorigenesis, suppression of cytotoxic T cell priming and function (16, 31–34), and T cell exhaustion (29, 35, 36). However, favorable TILs also exist in the TiME such as CD4+ and CD8+ T cell infiltrates (17, 18, 37). Multiple reports have identified an association between higher degrees of CD4+ and CD8+ T cell infiltration with improved survival in PDAC (38–40). Furthermore, the spatial organization of these immune infiltrates impact survival (19, 38), suggesting that a deeper understanding of the architecture and spatial organization of immune cell populations within the PDAC TiME is required to better appreciate their clinical and translational relevance. Tertiary lymphoid structures (TLS) represent an essential component of this immune spatial organization and are organized aggregates of immune cells that arise in nonlymphoid tissue. A growing body of evidence link TLS formation to improved clinical outcomes in a variety of cancers (41–43). This review will define TLS anatomy, the clinical significance, and translational implications for PDAC TLS to establish a deeper understanding of opportunities to harness anti-tumoral immunity that can translate to improved patient outcomes.

## 2 Anatomy of tertiary lymphoid structures

TLS have been identified in multiple inflammatory disease states (44) such as rheumatoid arthritis (45), Sjogren’s disease (46), autoimmune diabetes (47), and various cancers (43). In contrast to lymph nodes, TLS are non-encapsulated organized lymphocyte aggregates that can form in non-lymphoid tissues undergoing chronic inflammatory stress such as infection, transplantation, and cancer (48). In human malignancies, TLS generally contain the major constituents of adaptive immunity, with zones containing B cells, T cells, and a stromal network of follicular dendritic cells (FDCs) (49). High endothelial venules (HEVs) can also surround TLS to allow immune cell trafficking (50). Interestingly, in PDAC, small nerve fibers have been shown to exist in these aggregates and the density of such fibers has been associated with a prolonged overall survival (51).

**Figure 1:**
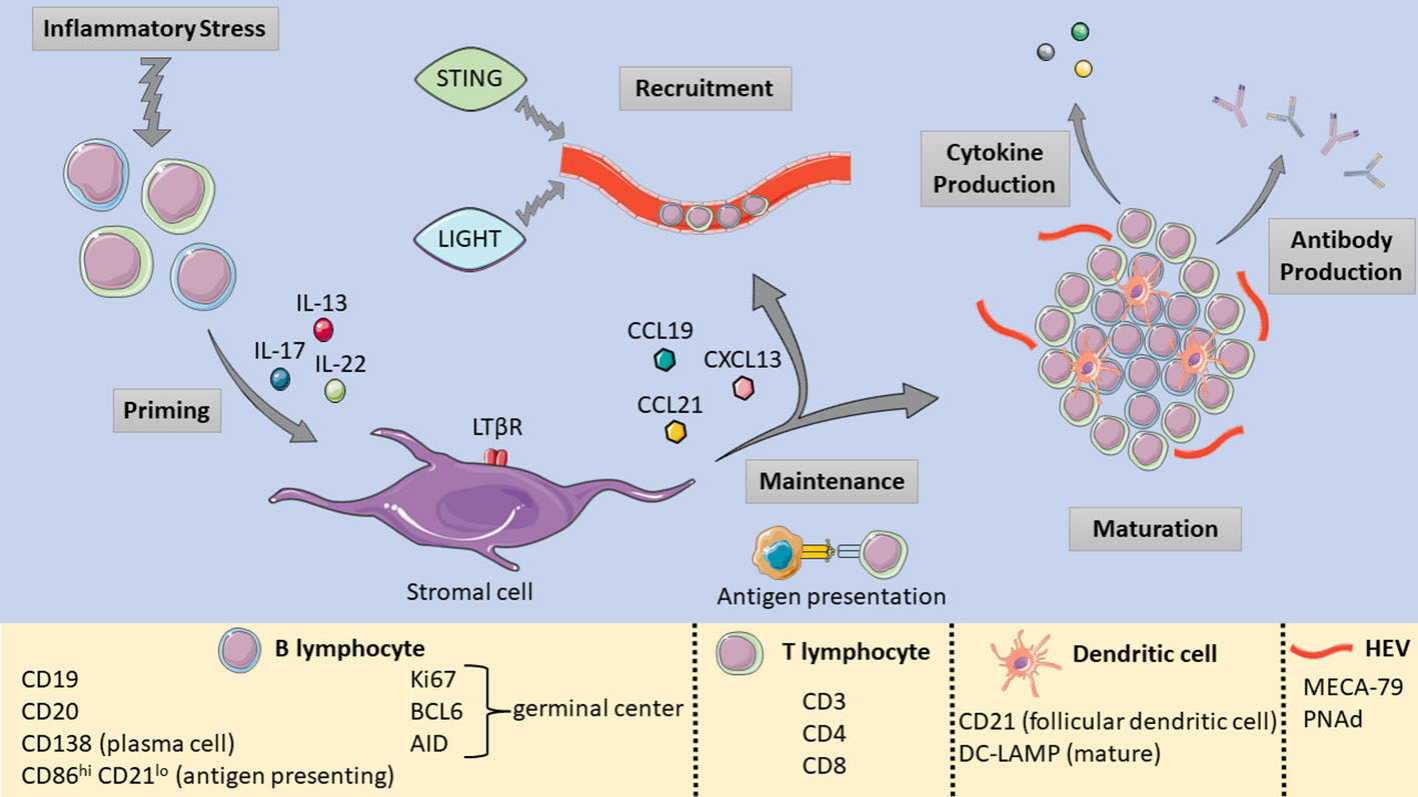
Pictorial summary of TLS formation, maturation, and the relevant markers used to identify TLS components. [Shapes and images are imported from Servier Medical Art by Servier (http://smart.servier.com/), accessed on May 19, 2023. Licensed under a Creative Commons Attribution 3.0 Unported License (https://creativecommons.org/licenses/by/3.0/)].

TLS formation can be broadly defined by three main phases: fibroblast activation, immune cell recruitment, and maturation (52) (**Figure 1**). Several cytokines such as IL-13, IL-17 and IL-22 are implicated in the initial priming of local fibroblasts by immune cells undergoing inflammatory stress (53, 54). At this stage, immature structures are formed, and their maintenance requires the retention of existing immune cells as well as the recruitment of further immune populations. Continuous local antigen presentation (55) and chemokines such as CXCL13 and others play a key role in these two processes respectively (56). If stressors persist, TLS maturation progresses with the formation of HEVs and germinal centers (GCs). Within PDAC, the B lymphocyte chemoattractant CXCL13 may be of particular relevance, as immunofluorescence studies of PDAC TLS demonstrate an abundance of CXCL13 distributed throughout TLS (57, 58). Furthermore, ectopic CXCL13 expression triggers TLS formation in the pancreas in both cancerous (59) and noncancerous (58) contexts. Other earlier studies also revealed the critical role of chemokines such as TCA4/SLC and CXCL13 (also known as B lymphocyte chemoattractant) (60) in pancreatic lymphoid neogenesis.

## 3 Mature and immature tertiary lymphoid structures

**Figure 2:**
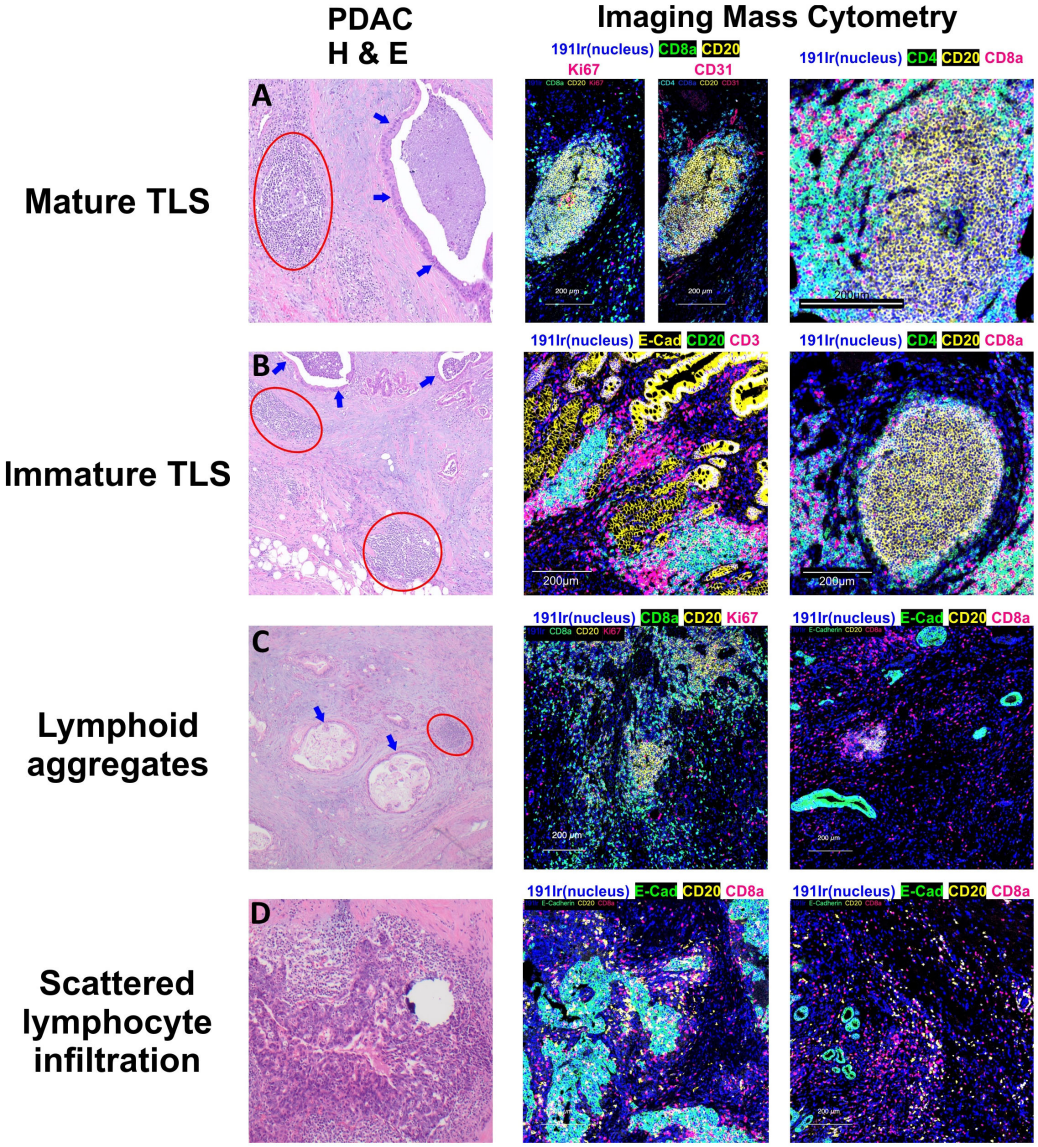
Patterns of Lymphocyte Infiltration in Pancreatic Adenocarcinoma. Tertiary lymphoid structures (TLS) in pancreatic adenocarcinoma display varying stages of maturation. **(A)** Mature TLSs comprise B cells, T cells, and follicular dendritic cells, and are distinguished by the presence of germinal centers containing Ki67(+) B cells. **(B)** In contrast, the majority of the observed TLSs are in an immature stage, lacking germinal centers but still exhibiting distinct B cell and T cell zones. **(C)** Apart from these structured aggregates, there are lymphoid aggregates which represent a disorganized accumulation of both B and T lymphocytes. **(D)** Moreover, individual B and T cells can also be observed dispersed within the tumor or surrounding the tumor cells. Circles in red show lymphoid structures, and arrows in blue display invasive adenocarcinoma with luminal necrosis.

The maturation from a loose aggregate of B cells and T cells into a mature, functional lymphoid structure impacts the TiME and carries broader clinical significance. TLS can undergo multiple stages of maturity (**Figure 2**), which have been defined by different criteria from different research groups. Earlier distinctions were typically made based on the presence of GCs within mature TLS compared to immature TLS, which were vaguely organized lymphocytic clusters without GCs (61, 62). More recently, one group defined three phenotypes of TLS maturity in a lung cancer model based on immune cell constituents and anatomical organization (63). Early TLS were dense lymphocytic clusters of CD21^−^ CD23^−^ B and T cells without FDCs or GCs localized near CXCL13-expressing perivascular cells, intermediate-stage or “primary follicle-like” TLS contained CD21^+^ CD23^−^ FDCs without GCs, and mature or “secondary follicle-like” TLS contained CD21^+^ CD23^+^ GCs in addition to the features of TLS in earlier stages of maturation. These maturity phenotypes were later validated in a colorectal cancer model from 109 patient-derived non-metastatic tumor specimens (64). Within studies of PDAC TLS, definitions of immature and mature TLS have varied between studies. Many groups defining PDAC TLS rely upon histological examination with hematoxylin and eosin staining to identify TLS with organized B cell and T cell zones, germinal centers, and HEVs, with immunohistochemical staining of surrogate markers such as peripheral lymph node addressin (PNAd) or Ki67 implying TLS maturity (65, 66), but do not directly identify early or intermediate TLS in comparison to mature TLS. Others fully characterize TLS based on distinct CD3+ and CD20+ B cell and CD8+ T cell zones with CD21+ FDCs and (PNAd+) HEVs (59, 67), providing a more specific definition of PDAC TLS maturity.

GCs are an essential component of peripheral lymphoid organs and important sites of antigen-driven somatic hypermutation and memory B cell and plasma cell maturation (68). Similarly, the GCs of mature TLS contain proliferating B cells and DC-LAMP^+^ FDCs (69), characterized by Ki67, activation-induced cytidine deaminase (AID) and BCL6 expression (67, 70). HEVs are also present in these structures and are classically identified by the presence of an L-Selectin ligand, MECA-79, and PNAd (71). These HEVs play an essential role in mature TLS function by mediating immune cell trafficking (72) via binding of L-Selectin to PNAd (73).

Evidence from diabetic mice with autoimmune insulitis revealed that functional and mature TLS within the pancreas can harbor differentiated autoreactive B cells that express the activation-induced cytidine deaminase (AID) enzyme which mediates immunoglobulin class switching and affinity maturation (70). As with other neoplasms, expression of Ki67, BCL6 and CD21 in B cell zones is indicative of mature PDAC TLS, with GCs containing B cells undergoing affinity maturation and somatic hypermutation (67). In murine PDAC models, the presence of HEVs in mature TLS have been shown to facilitate lymphocytic infiltration (74) and enhance their activation via LTβR signaling (75).

However, TLS in cancers exhibit significant heterogeneity in maturation, and can also vary in maturity depending on tumor stage (76) or metastatic site (77). PDAC may present a unique challenge in understanding TLS maturity, as the PDAC TiME exhibits considerable immune heterogeneity (78, 79) and lower proportions of TLS are found in PDAC compared to other cancers (43). Relatively few of TLS found in PDAC are mature and possess GCs (67). A timeline of TLS maturity has been proposed (63). Although it remains unclear whether the various phenotypes in PDAC represent a stepwise progression of maturation or are instead terminally differentiated and unable to eventually transform into mature TLS. Understanding these nuances may improve both our knowledge of the PDAC TLS as well as broaden our understanding of the PDAC TiME as a whole.

## 4 Location and density

TLS can be located either intratumoral or peritumoral. TLS location has significant anatomical and functional implications. In cancers such as melanoma, increased peritumoral mature TLS density is associated with longer survival (74). PDAC TLS formation is more abundant in the invasive front of the tumor and peritumorally compared to the tumor center (80). One study conducted on more than 300 PDAC human samples demonstrated that 84% of the examined samples possessed only peritumoral TLS, while only 16% contained intratumoral TLS (65). Interestingly, a more recent study demonstrated that despite being less plentiful, the intratumoral TLS had a greater B and T cell infiltration, less immunosuppressive populations, a significant Th1 and Th17 pro-inflammatory genetic polarization, and were associated with improved survival (65). The intratumoral TLS were also more mature with intact nerve networks and organized vasculature formed by mature endothelial cells circled by pericytes. PDAC is characterized by a desmoplastic, fibrotic stroma (81–83), which may necessitate a spatial adjacency of TLS to tumor for its anti-tumoral effects to manifest.

## 5 Relevant cell populations within PDAC TLS

As TLS share morphological and cellular similarities with traditional secondary lymphoid organs, they may also recapitulate some of their functionality. B cells are an essential component of TLS and are present at all stages of TLS maturity (66, 69). Data from the study of TLS in autoimmune disease support the idea that B cells within the TLS GC undergo somatic hypermutation and clonal expansion in rheumatoid arthritis (84), Sjogren’s disease (85), myasthenia gravis (86), and autoimmune diabetes. TLS-associated B cells are also capable of terminal differentiation into antibody-secreting plasma cells (87). Similar to the data from autoimmune TLS research, cancer TLS-associated B cells are proliferative, experience somatic hypermutation, and undergo maturation into antibody-secreting plasma cells (69, 88). These plasma cells also produce tumor-specific antibodies (69, 88, 89) capable of binding to tumor cells and enhancing immunotherapy (88), suggesting that the TLS B cell population fulfills an antitumoral niche within the more generalized B cell infiltrate in tumors.

Data from retrospective studies and preclinical models in PDAC highlight the antitumoral function of the TLS-associated B cell population. One group showed that TLS+ PDAC tumors have a higher proportion of memory B cells and memory IgG1 class-switched B cells compared to TLS- tumors (67), while another group demonstrated that TLS-associated B cell infiltrates in a KPC PDAC murine model exhibited an immunostimulatory phenotype, with upregulation of proinflammatory (SPP1, IL6, CSF2, VEGFA, CCL4, PTGS2) and T cell chemotaxis (CXCL1, CXCL2, CXCL5, CCL2, CXCL12, CCL20) genes, along with downregulation of immunosuppressive genes (CD274 and IL12a) (90, 91).

T cells are an essential component of the TLS (49). As higher CD8 T cell tumor infiltration is associated with increased survival across cancers (92–94), understanding the interaction of CD8 T cell populations and TLS is of particular interest. Preclinical autoimmune diabetes models have linked TLS with naïve T cell recruitment and proliferation (95). Within a cancer-specific context, LIGHT expression in a fibrosarcoma model induced the formation of TLS, naïve T cell recruitment, T cell proliferation and priming, and subsequent tumor regression (96). A preclinical colorectal cancer model showed that TLS were associated with CD3+ T cell infiltration and mediated GFP+ splenocyte recruitment (97), and a preclinical melanoma model demonstrated an association between TLS and tumor-specific T cell responses independent of secondary lymphoid tissue (98).

Multiple retrospective studies from human PDAC samples show an association between TLS formation and increased CD8 T cell infiltration (91, 99, 100). For example, evidence from one group demonstrated that T cells were enriched in mature TLS with PNAd+ HEVs, TLS presence correlated with greater intratumoral and circulation CD8 T cell populations, and the TLS+ tumor stroma had a higher CD8/Treg ratio (100). Based on a PDAC tissue microarray, another investigation identified a “TLS rich” immune PDAC subtype with higher expression of T cells (CD8, CD3, and CD4) and B cells (CD20) markers and lower in Treg (FOXP3) markers (101).

The mechanistic interactions between CD8 T cells and the PDAC TLS in PDAC are just beginning to be explored and suggest that PDAC TLS are active participants in T cell maturation and trafficking. Naïve T cell infiltration appears dependent upon PNAd+ CCL21+ HEVs seen in mature TLS (102). Data from a tumor vaccine-induced human PDAC TLS cohort demonstrated high expression of the early T cell activation marker CD69 and T cell trafficking receptor CXCR3 within TLS, implicating their role in T cell activation (103). Furthermore, Vβ2-positive T cells are present in the center of PDAC TLS, suggesting that TLS are sites of T cell clonal expansion (104).

Additional anti-tumoral efficacy may be related to T follicular helper cells (Tfh), which are a component of TLS (105) associated with improved prognosis in cancers (43, 106, 107). PDAC Tfh from patients who received neoadjuvant chemotherapy exhibited increased CD8 T cell and B cell recruitment capability compared to treatment-naïve Tfh (108).

Interestingly, cancer-associated fibroblasts (CAFs) may also play a role in TLS function. PDAC CAFs are typically associated with tumorigenic (109) and immunosuppressive roles (110). However, diverse CAF subpopulations (111, 112) exist within PDAC. Nonspecific ablation of CAFs induces both immune remodeling and a more aggressive tumor phenotype (113, 114), suggesting that an immunologically favorable and tumor suppressive CAF subpopulation exists within PDAC. Similar to other cancers, where CAF subtypes can promote TLS formation (115), a link between PDAC CAFs and PDAC TLS may exist. A recent study described associations between CAF-derived TGF-β with increased TLS gene signatures within PDAC (57). Furthermore, the authors observed that the expression of CXCL13 on CD4 and CD8 T cells occurred in a TGF-β dependent fashion, highlighting the interaction between PDAC TLS and CAFs.

However, this immunologically favorable CAF-TLS interaction in PDAC is still relatively undefined. For one, if the immunologically favorable CAF subtype exists, it is not fully characterized. A potential biomarker of this putative CAF subtype may be podoplanin (PDPN), a lymphatic endothelial and CAF marker (116, 117). PDPN expression on fibroblasts can promote TLS establishment (54), while recent studies in PDAC demonstrated that a PDPN+ CAF subtype (112, 118) was associated with improved prognosis and enrichment of immune-related pathways, However, the role of PDPN in cancer is controversial, and associated with worse outcomes in multiple cancers, including PDAC (119, 120). A more thorough understanding of CAFs and CAF plasticity in PDAC (120) may provide clarification. 6 Clinical significance of PDAC TLS.

In a variety of cancers, mature TLS are associated with improved progression-free survival (41–43, 63, 64, 121) and response to immunotherapy (122). Similarly, a preponderance of data from human PDAC samples have linked TLS with improved survival (**Table 1**) (65, 67, 91, 99–101, 123, 124). Multiple groups demonstrated a survival benefit with the presence of mature TLS (98, 100, 103). Other have reported the prognostic presence of with intratumoral TLS (123). One of the first groups to report on TLS in PDAC examined surgical specimens from 308 treatment-naïve PDAC patients and identified mature TLS based on histopathological examination (65). Within their study cohort and an additional validation cohort of 226 patients, 84% of samples contained peritumoral TLS, while 16% contained intratumoral TLS. The presence of intratumoral TLS conferred a significant survival benefit, with a median survival of 42.7 months compared to 29.4 months for those with only peritumoral TLS. The presence of intratumoral TLS also correlated with increased CD4 and CD8 T cell infiltration and decreased Treg and M2 macrophage infiltration in the TiME, suggestive of a more anti-tumoral and immunogenic TiME. These findings are corroborated by other retrospective studies examining TLS in surgical PDAC specimens (67, 99, 101).

**Table 1:** Retrospective analyses of TLS in PDAC and survival outcomes.

Cancer Types	PDAC Stage	# of Patients	TLS Findings	Outcome Measure	Reference
PDAC only	Resectable	534	TLS Grade (based on localization and frequency): grade 1 (49%), grade 2 (35%), grade 3 (14.3%), and grade 4 (1.6%)	mOS (42.7months (intratumor TLS+) vs 15.5 months (TLSneg); p < 0.05)	(65) Hiraoka, N., et al. British Journal of Cancer, 2015. 112(11): p. 1782-1790.
PDAC only	All	140; surgery alone n= 93; NAC n=47	TLO rate (n=128/140) 91.4%; no significant difference between surgery alone and NAC (p=0.058)	5-year OS: 44.2% (NAC) vs 17.7% (surgery alone); p = 0.0017. In NAC group: high TLO/tumor ratio had better prognosis than low TLS/tumor; p = 0.0326	(66) Kuwabara et al. Cancer Science, 2019. 110(6): p. 1853-1862.
PDAC only	Resectable	63	TLS + in n=29 (46%) with at least 2 organized lymphoid aggregates.	mOS (26.3 in TLS+ vs 14.4 months in TLSneg; p = 0.014, HR 1.96)	(67) Gunderson, Andrew J., et al. Oncoimmunology, 2021. 10(1): 1900635.
PDAC only	Resectable	104	CD20-TLT immune reactive area (IRA%) range: 0-23.5% and CD20-TIL IRA% range: <0.05-1.89%	immune signature comprising CD20-TLT^hi^/CD20-TIL^lo^ with mOS 30.9mo versus 14.1mo (other combos); p = 0.0051	(91) Castino et al. Oncoimmunology, 2016. 5(4): p. e1085147
PDAC only	Resectable	55	TSL + in n=38 (69%)	TLS + had improved OS compared to TLS neg (HR 0.509, 95% CI (0.29-0.89); p = 0.018	(99) Ahmed et al. Oncoimmunology, 2022. 11(1): p. 2027148.
PDAC and pNET	Resectable	27; n=20 PDAC, n=7 pNET	CD20+ TLS in 64% (9/14 PDAC) in tumor stroma (no NAT); TLS infrequent in pNET and rare in PDAC who received NAT (1/6).	mOS TLS+, 755 days; TLSneg, 478 days; HR 0.15, 95% CI, 0.02-1.19, P = 0.07)	(100) Stromnes et al. Cancer Immunology Research, 2017. 5(11): p. 978-991.
PDAC	Resectable	110	TLT presence (17.5% of PDAC) correlated with low-tumor budding and increased OS (p<0.0001) and increased DFS (p = 0.0067)	mOS of Immune-rich with TLTs of 23mo vs. 10mo for immune-escape	(101) Wartenberg et al. Clinical Cancer Research, 2018. 24(18): p. 4444-4454
PDAC	Resectable	127	75% samples had intermediate (100–300 CD3+ cells/mm2) or high (>300 CD3+ cells/mm2) infiltrating T-cells	mOS for TLS+ (n=76) of 27.5mo vs. 14.6mo (n=51) for TLSneg; p=0.0284	(104) Poschke et al. Oncoimmunology, 2016. 5(12): p. e1240859.
PDAC	Resectable	n=380 without NAT; n=136 with NAT	TLS in 80/380 (21.1%) of surgery alone and 21/136 (15.4%) with NAT	Surgery alone: TLS+ had improved OS 24mo vs 12mo in TLSneg p=0.0011 NAT: no significant difference in OS based on TLS.	(123) Xuan et al. Journal for ImmunoTherapy of Cancer, 2023. **11**(6): p. e006698.
PDAC only	Resectable	162	TLS+ in n=112 (69.1%)	Pancreatic cancer-specific survival with adjuvant S-1: HR 0.37; 95% CI 0.25 - 0.56; p < 0.0001	(124) Tanaka et al. Journal of Gastroenterology, 2023. 58(3): p. 277-291
Lung, sarcoma, bladder, colorectal, head/neck, renal, breast, PDAC	All	328	mature TLS positive: 84 cases (25.6%)	anti-PD1 or anti-PDL1 treatment: PFS (6.1 vs 2.7 months; p = 0.015), mOS (24.8 vs 13.3 months; p = 0.016)	(124) Vanhersecke et al. Nature Cancer, 2021. 2(8): p. 794-802.

*NAC, neoadjuvant chemotherapy; TLO, tertiary lymphoid organ; NAT, neoadjuvant therapy; TLS, tertiary lymphoid structures; TLT, tertiary lymphoid tissue; TIL, tumor infiltrating lymphocytes.

Importantly, many of these studies describing mature TLS are based upon histological examination and identification of TLS based on the presence of GCs and surrogate markers of maturity such as PNAd+ HEVs (65, 93, 101, 124) and are likely excluding early and immature TLS from analysis. Precise characterization of TLS maturity may have clinical ramifications, as one study reports early PDAC TLS having greater CD8+ T cell infiltration and being enriched for IgG1 class-switched memory B cells and memory CD4+ T cells (67). Using the Cancer Genome Atlas Program PDAC datasets, early TLS are found to harbor decreased tumor molecular burden, but mature TLS expressed more neoantigens and increased B cell somatic hypermutation (78).

Aside from their impact on CD8 T cells, mature TLS with GCs were enriched for other immunologically relevant immune cell phenotypes, including activated CD4+ memory cells, naïve B cells, and NK cells (67). The interaction between TLS and B cells is noteworthy; in cancers, the role of B cell infiltration is controversial, and has been linked to both pro-tumorigenic (125–127) and anti-tumorigenic (69, 91, 97) states. In human PDAC samples, the spatial distribution of B cells is predominantly within either TLS or at the tumor-stromal interface (91). Interestingly, only TLS-associated B cells were associated with improved prognosis, while an immune signature associated with increased TLS-associated B cells and low B cell TILs predicted longer overall survival (91), supporting the clinical relevance of a TLS-specific subset of B cells within PDAC.

## 6 Preclinical evidence for targeting TLS

As TLS are associated with favorable TiME characteristics such as an increased level of CD8+ TILs (43, 128), targeting PDAC TLS may improve patient outcomes. Multiple studies (129–132) support the claim that standard of care chemotherapy can favorably alter the PDAC TiME to create an anti-tumoral immune niche. Evidence from matched pre- and post-neoadjuvant treated APC germline mutated hepatoblastoma samples suggested that cisplatin can induced immature TLS formation (133). Within PDAC, a preclinical TLS model established that TLS formation can be induced after intratumor injections of chemokines CXCL13 and CCL21 in an orthotopic pancreatic cancer mouse model (60). Furthermore, coadministration of gemcitabine with these lymphoid chemokines led to significant tumor reduction (60). It should be noted that the sole administration of gemcitabine within this study globally reduced immune cell infiltration within the tumors, while combination chemokine and gemcitabine therapy ameliorated this effect.

Vaccine-based PDAC immunotherapy trials in PDAC also highlight the role of TLS in immunotherapy. Vaccinating mice that spontaneously develop PDAC Kras^G12D^ Pdx1-Cre (KPC) tumors with an α-enolase (ENO1) encoding vector led to a spatial reorganization of the TiME that is marked by TLS induction. These TLS had a higher number of PD-1^+^ germinal center and vaccinated mice had an ameliorated infiltration of antigen-specific T cells (91). A randomized clinical trial with administration of a granulocyte-macrophage colony-stimulating factor (GM-CSF)-allogeneic pancreatic tumor cell vaccine (GVAX) demonstrated the induction of mature TLS formation alone or in combination with low dose cyclophosphamide (134). These vaccine-induced TLS possessed multiple antitumoral attributes such as effector T cell trafficking, T cell activation, and differential expression of immune-regulating pathways. These induced TLS also expressed PD-1 and PD-L1, suggesting the benefit of combination with ICI therapy.

## 7 PDAC TLS and therapy response

TLS presence is associated with neoadjuvant therapy response in cancers (96, 135, 136). Data on TLS and response to chemotherapy in PDAC remains limited. One group compared a total of 140 resected PDAC specimens from patients who received up-front surgery to those who received neoadjuvant therapy. The TLS from the neoadjuvant therapy group possessed higher proportions of CD8 T cells and lower proportions of PD-1 expressing lymphocytes compared to those from the up-front surgery group, suggesting the induction of an antitumoral niche within TLS through neoadjuvant therapy (66). Another retrospective analysis of 162 patients found that PDAC patients with TLS had longer cancer-specific survival when treated with 5FU-based therapy compared to gemcitabine-based or no adjuvant therapy (124). As platinum-based therapies such as cisplatin and oxaliplatin can interact with the TiME by inducing cytotoxic T cell activity through immunogenic cell death (137–140), the choice of chemotherapy may impact the predictive value of TLS. In contrast, a recent study of 380 treatment-naïve PDAC patients and 136 PDAC patients treated with predominantly gemcitabine-based neoadjuvant chemotherapy demonstrated significant alterations of the neoadjuvant-treated intratumoral TLS (123). The neoadjuvant-treated samples possessed fewer intratumoral TLS compared to the treatment-naïve samples, and the TLS within the neoadjuvant-treated group had significantly lower B cell proportions and higher Treg and macrophage proportions compared to their untreated counterparts (123). Furthermore, in contrast to the treatment-naïve group, the presence of intratumoral TLS within the neoadjuvant-treated group was no longer significantly associated with survival, suggesting that interactions between PDAC TLS and chemotherapy may depend on multiple factors such as the choice of chemotherapy, duration of therapy, or initiation with adjuncts such as corticosteroids (141).

Recent studies have also evaluated the favorable interaction between TLS and immunotherapy across a spectrum of cancers (142–144). As PDAC responds poorly to immune checkpoint inhibitors (ICIs) (9, 10), understanding the anti-tumoral and ICI-potentiating benefit of PDAC TLS becomes critical to improving treatment efficacy. Data from a cohort of 328 patients treated with immunotherapy, including a subset of patients diagnosed with PDAC, identified that mature TLS presence was correlated with increased CD8 T cell density, was predictive of response to ICIs, and an independent predictor of progression free and overall survival (122). These findings also suggest TLS may serve as marker for favorable outcome with FOLFIRINOX and can identify patients for future chemo-immunotherapy trials.

Similarly, as data are beginning to evolve regarding the role of TLS with immunotherapy responses in several aggressive cancer types, a number of clinical trials assessing immunotherapy as an effective treatment strategy for pancreatic ductal adenocarcinoma have been conducted (NCT03214250, NCT03404960, NCT02879318, NCT01836432). However, no trials have also investigated the role of immunotherapy with induction or presence of TLS or in respect to other modulations of the TiME in PDAC. This underscores the need for additional translational studies investigating the role of both TLS and TiME as they relate to immunotherapy responses in PDAC.

## 8 Future directions to enhance TLS function

As our understanding of the role of TLS in cancers improves, the translational utility of manipulating TLS has become an area of interest as well. As an initial step within the clinical setting, several well-known markers could be used to identify TLS. These could include CD3, CD4 and CD8 for T cells, CD19 or CD20 for B cells, CD21 for FDCs, CD138 for plasma cells, DC-LAMP for mature FDCs, as well as other activation markers (48). Antigen presenting B cells with a high expression of CD86 and a low expression of CD21 have been shown to localize in TLS in several cancers (145).

A chemokine-based “12-CK score” (CXCL9, CXCL10, CXCL11, CXCL13, CCL2, CCL3, CCL4, CCL5, CCL8, CCL18, CCL19 and CCL21) was initially proposed and developed based on colorectal cancer samples (146). Subsequently, this score has been validated as a TLS transcriptomic signature with prognostic significance in several neoplasms (147). A similar 12-chemokine gene signature (CCL2, CCL3, CCL4, CCL5, CCL8, CCL18, CCL19, CCL21, CXCL9, CXCL10, CXCL11, and CXCL13) was derived through metagene analysis and correlated with enhanced patient survival in colorectal cancer (146) independent of tumor stage, location, microsatellite stability status, or treatment. As a single marker, IL-2 levels could be used as a potential biomarker for PDAC TLS formation, as analysis of paired blood and tumor samples from PDAC patients revealed the existence of a “stroma-to-serum” gradient in patients that lack TLS and an association between lower serum IL-2 levels and TLS formation (99). These findings highlight the potential of using serum biomarkers to predict response to immunotherapy; however, additional work needs to be done to determine the optimal time of IL2 monitoring.

Looking ahead, preclinical and clinical studies of the molecular manipulation of TLS may inform PDAC TLS strategies. For example, Tregs play an important role in TLS maintenance and can dampen anti-tumor responses in secondary lymphoid organs, e.g., lymph nodes and spleen. Tregs express high amounts of IL2 receptor as well as immunoinhibitory receptor for PD-1. Infiltration of Tregs into lung adenocarcinoma mouse models was found to suppress anti-tumor responses in TLS; ablation of Tregs induces strong effector T cell responses and tumor destruction (148, 149). Treg deletion may be another promising therapeutic option.

Lymphotoxins belong to the tumor necrosis factor (TNF) superfamily and play an important role in lymphoid tissue organogenesis and may be associated with TLS development (150). Lymphotoxin beta receptor (LTβR) signaling has been shown to mediate the formation of HEVs (72) and FDCs (151). Another lymphotoxin, LIGHT/TNFSF24, increases lymphoid penetration and lead to changes in the TIME, such as the development of TLS (152, 153). Evidence from more than two decades ago revealed that the ectopic expression of chemokines like CCL19 and CCL21 in pancreatic islets led to small-sized infiltrates of lymphocytes with stromal cells and HEVs (154, 155). Other earlier studies also revealed the critical role of chemokines such as TCA4/SLC (60) in pancreatic lymphoid neogenesis. In an orthotopic murine mesothelioma model expressing human mesothelin, treatment with a mesothelin-targeted Fab linked to a toxin eradicated tumors and induced TLS formation (156). Knowing that mesothelin is expressed in almost all pancreatic ductal adenocarcinomas (157), its potential role as a therapeutic target to induce TLS formation in PDAC warrants further investigation.

Looking toward evidence from other preclinical cancer models, other methods to induce TLS formation may be applicable to PDAC. Stimulator of interferon genes, or STING, is a double-stranded DNA sensor, that along with STING agonists, can activate antitumoral immune responses (158). One group demonstrated that intratumoral injection of ADU S-100, a STING agonist, in a preclinical melanoma model promoted formation of CCL19+, CCL21+, PNAd+ TLS, although these TLS did not contain significant B cell infiltration or GCs (159). Similarly, oral administration of an oncolytic virus (160) carrying a vector expressing IL-15 produced similar results in lung cancer and melanoma mouse models (161). This is intriguing not only for the potential of inducing PDAC TLS formation through delivery of lymphogenic cytokines, but the use of oncolytic viruses in PDAC (162) may serve as an effective method to overcome barriers to drug delivery in PDAC (134, 163). This could also be adapted to potentiate the function of existing TLS by inducing immunogenic cell death (164) to enhance T cell mediated immune responses that may originate from pre-existing PDAC TLS.

Other future strategies to potentiate PDAC TLS function may target specific cell populations found within the TLS. The PDAC immune microenvironment possesses immune checkpoint heterogeneity (17), with a distinct subset of exhausted CD8 TILs expressing T-cell immunoglobulin and ITIM domain (TIGIT) (165). A recent study demonstrated the presence of CD8 T cells expressing both PD-1 and TIGIT which were located predominantly within PDAC TLS (166). PD-1+ TIGIT+ Cd8 T cells are associated with worse prognosis in cancer (167), and the relationship between this dual immune checkpoint expressing T cell phenotype and the PDAC TLS have yet to be elucidated. However, dual checkpoint blockade against PD-1 and TIGIT partially restored T cell proliferation and cytokine production (166), suggesting a potential therapeutic avenue for potentiating a TLS-mediated T cell response. CAF-specific therapy strategies targeting CAF plasticity may also be of potential benefit. Multiple phenotypically diverse CAF subtypes have been described in PDAC (112, 116, 118, 121). While the majority of CAF subtypes possess tumorigenic and immunosuppressive capabilities, evidence suggests that a minority of PDAC CAFs are associated with upregulation of immune signatures and TLS formation (57, 112, 118). Strategies involving CAF reprogramming (168, 169) may provide a useful adjunct to TLS-focused strategies in the future.

## 9 Conclusions

As our understanding of PDAC TLS continues to evolve, it is becoming increasingly evident that they hold promise as a prognostic marker and therapeutic target. The manipulation of TLS may pave the way for novel diagnostic tools, innovative immunotherapeutic strategies, combination treatments, and ultimately improving clinical outcomes for a patient population in need of improved outcomes. Moving forward, further research and clinical trials are warranted to capitalize on the potential of TLS as an integral element in the fight against PDAC.

## Author contributions

ZG: Conceptualization, Writing – original draft, Writing – review & editing. JA: Visualization, Writing – review & editing. HZ: Writing – review & editing. SW-P: Writing – review & editing. SK: Data curation, Visualization, Writing – review & editing. CM: Visualization, Writing – review & editing. MR: Writing – review & editing. SM: Writing – review & editing. H-SL: Visualization, Writing – review & editing. EC: Writing – review & editing, Funding acquisition, Project administration, Supervision.
